# Nanomechanical Molecular Mass Sensing Using Suspended Microchannel Resonators

**DOI:** 10.3390/s21103337

**Published:** 2021-05-11

**Authors:** Alberto Martín-Pérez, Daniel Ramos, Javier Tamayo, Montserrat Calleja

**Affiliations:** Bionanomechanics Lab, Instituto de Micro y Nanotecnología, IMN-CNM (CSIC), Isaac Newton 8 (PTM), E-28760 Tres Cantos, Madrid, Spain; alberto.martin@csic.es (A.M.-P.); jtamayo@imm.cnm.csic.es (J.T.); montserrat.calleja@csic.es (M.C.)

**Keywords:** microcapillary, transparent resonators, interferometry, optomechanics, microfluidics, gas sensing

## Abstract

In this work we study the different phenomena taking place when a hydrostatic pressure is applied in the inner fluid of a suspended microchannel resonator. Additionally to pressure-induced stiffness terms, we have theoretically predicted and experimentally demonstrated that the pressure also induces mass effects which depend on both the applied pressure and the fluid properties. We have used these phenomena to characterize the frequency response of the device as a function of the fluid compressibility and molecular masses of different fluids ranging from liquids to gases. The proposed device in this work can measure the mass density of an unknown liquid sample with a resolution of 0.7 µg/mL and perform gas mixtures characterization by measuring its average molecular mass with a resolution of 0.01 atomic mass units.

## 1. Introduction

Detecting the presence of pollutants either in air or water at very low concentrations is a key point in a wide range of fields ranging from the environmental control [1,2] to safety in the food industry [3]. Conventional techniques employed for this detection are based on specific recognition of certain pollutants offering high-sensitive detection. However, they may be limited to a particular pollutant and multiplexing can be troublesome. In addition to this, the recognition process saturates the sensor surface, diminishing its life-time. To overcome this problem, physical parameter-based sensors have been proposed as a promising alternative. They have been used for non-selective fluid discerning [4] and characterization of density [5], refractive index [6,7] or compressibility [8,9] of analytes in a liquid environment. By using these physical sensors, it is also possible to track in real-time the changes in the physical properties of fluid mixtures with varying concentrations of their components [10,11,12,13].

In this regard, suspended microchannel resonators (SMR) have been demonstrated to be excellent tools for highly sensitive detection of both colloidal particles (measuring its buoyant mass) [14,15,16,17] and liquid properties (by measuring its mass density, viscosity, etc.) [18,19,20,21,22]. Among other nanomechanical techniques, SMR approach is characterized for placing a liquid inside the resonator while vibrating in vacuum or gaseous atmosphere. Under these conditions, the dragging forces acting on the resonator are minimized showing outstanding resolutions of 10 ag for particle mass and 2.5 µg/mL for fluid mass density in the state-of-the-art sensors [23,24]. Nevertheless, measuring density by itself does not allow to unambiguously discern between fluid mixtures, since different mixtures of different compounds may present the same density depending on its concentration. This problem becomes even more dramatic for gases, as their density is not as well defined as in liquids due to their compressibility. To overcome this problem, transparent microcapillary resonators (TMR) have been developed to combine the highly sensitive mass density sensing, measured from its mechanical frequency signal [25,26,27,28], with refractive index measurements, obtained from its reflectivity signal with detection limit of 10^−5^ [7,29].

In this work we study the effects of the hydrostatic pressure of the inner fluid on the resonance frequency for a transparent microcapillary resonator (TMR), demonstrating that this frequency shift is directly related to the physical parameters of the fluid such as mass density, compressibility, and molecular mass. To this purpose, we propose an analytical model, which is corroborated by finite elements simulations and experimental measurements, to calculate the fluid density and compressibility by means of the measurement of the resonant frequency of the TMR. We have attained a density resolution of 0.7 µg/mL when working with aqueous solutions, one order of magnitude better than current state-of-the-art, and a molecular mass resolution of 0.01 Da for gases. These results open the door for the characterization of fluids through the measurement of their molecular masses by using suspended microchannel resonators.

## 2. Experimental Setup

**Figure 1:**
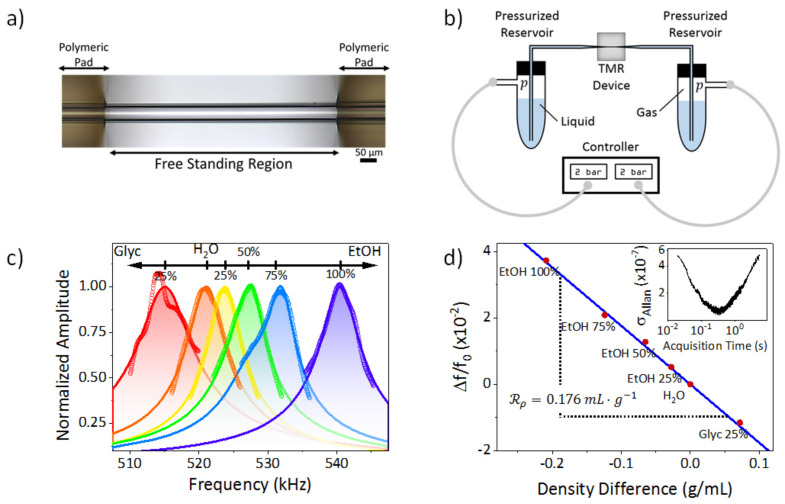
Experimental setup. (**a**) Optical microscopy image of the transparent microcapillary resonator used in the experiments; (**b**) Schematic of the pressurized reservoirs and their connection to the TMR device. The liquid can be removed so as to fill the device with gas; (**c**) Oscillation amplitude as a function of the excitacion frequency (Mechanical spectra) for the fundamental mechanical mode of the TMR device measured when filled with fluids of different densities (points) and their fittings to a harmonic oscillator model (solid lines) for different aqueous solution; (**d**) Calibration curve: frequency shift as a function of density, the slope of this curve is the density responsivity. Inset. Allan variance as a function of the acquisition time.

We have fabricated a transparent microcapillary resonator based on a thermally elongated silica capillary tube. The elongated region is integrated on a silicon chip and doubly-clamped by photolithographed polymeric pads (SU-8), obtaining a 30 µm outer diameter, 23 µm inner diameter and 500 µm long free-standing tube (Figure 1a) which can oscillate in a guitar string mode (fundamental mechanical resonance of 519 kHz). The first flexural mode of this suspended microchannel is excited by means of a piezoelectric device while its resonance frequency is real-time tracked by means of a home-made interferometric readout system and analyzed by a lock-in amplifier (HF2LI, Zürich Instruments AG, Zürich, Switzerland). Further details about fabrication and readout system can be found in the literature [7,29,30]. Both ends of the TMR device are connected to pressurized reservoirs containing a fluidic sample to be pumped into the device (either liquid or gaseous, Figure 1b). These reservoirs are pressurized by introducing a gas by means of a pressure controller (Fluigent INC, MFCS-EZ-07000001, Le Kremlin-Bicêtre, France) so we can apply a hydrostatic pressure over the atmospheric pressure when both reservoirs have the same gas pressure [31]. Please note that when working with liquid samples the carrier gas (usually nitrogen) transmits its pressure to the liquid so liquid and carrier gas pressure must be the same.

This experimental setup allows measuring pressure-induced mass density variations. The free-standing region of the resonator consists on a fused silica wall (constant mass, $m_{w}$) and an inner volume ($V_{in}$) which can be filled with different fluids (variable mass). Therefore, the mass of the resonator, and consequently its resonance frequency, accordingly vary with the fluid density. We define density responsivity as the variation of the normalized resonance frequency shift as a function of the fluid density. By calculating the resonance frequency from the Euler-Bernouilli beam theory [32] (Equation (1)), density responsivity ($R_{\rho }$) can be written as shown in Equation (2).

(1) $$f_{n}=\frac{\alpha_{n}{}^{2}}{4\pi }\sqrt{\frac{E\left(R_{out}{}^{4}-R_{in}{}^{4}\right)}{L^{3}\left(m_{w}+\rho V_{in}\right)}}$$

(2) $$R_{\rho }=\left|\frac{\partial \left(\Delta f/f_{0}\right)}{\partial \rho }\right|=\frac{1}{2\rho \left(1+\frac{m_{w}}{\rho V_{in}}\right)}\;$$

with $\alpha_{n}$ being the nth mode eigenvalue (first four eigenvalues, 4.7300, 7.8532, 10.9956, 14.1372…), E wall’s Young modulus, $R_{out}$ the outer radius, $R_{in}$ the inner radius, L the suspended length, ρ the fluid density and $\Delta f/f_{0}$ the normalized resonance frequency.

The calibration of our TMR device is made by measuring its resonance frequency filled with different aqueous solutions of ethanol (EtOH) and glycerol (Glyc) with different volumetric concentrations of well-known densities [10,11] (Figure 1c). The resonance frequency variation as a function of fluid density presents a linear dependency (Figure 1d), with responsivity of 0.176 mL·g^−1^, which is in good agreement with the value predicted by Equation (2), (0.184 mL·g^−1^). Combining this responsivity with the minimum value of the Allan variance, 1.3 × 10^−7^ (Figure 1d, inset) we obtain a density resolution of 0.7 µg/mL when working with aqueous solutions, one order of magnitude better than the current state-of-the-art [24]. Given the resolution shown by the devices and the pressure range our pump can apply, we are able to measure the density variations caused by compressibility effects either in liquids (~10 µg·mL^−1^·bar^−1^) or gases (~100 µg·mL^−1^·bar^−1^).

Density responsivity must be different for aqueous solutions and gases since it depends not only on the device parameters but also on the fluid properties. Responsivity calculated from Equation (2) has a value of $R_{\rho ,gas}$ = 0.280 mL·g^−1^ for gases.

## 3. Analytical Model

**Figure 2:**
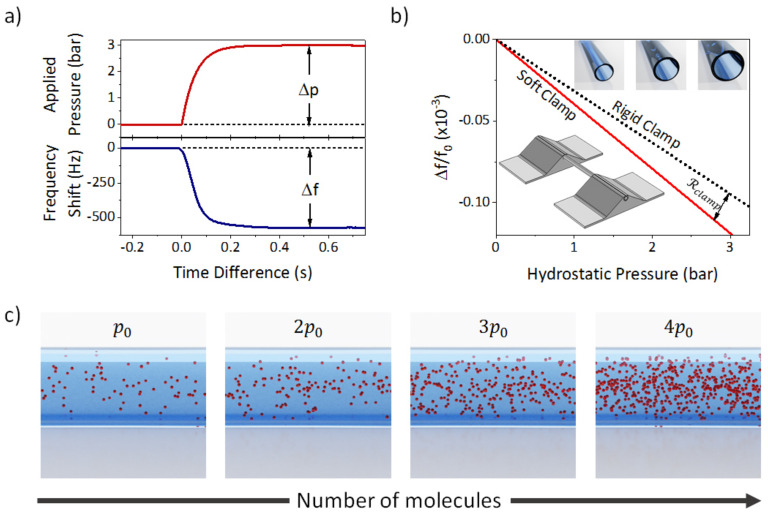
Pressure-induced changes in resonance frequency. (**a**) Experimental measurement of frequency shift and applied pressure in real time for the TMR filled with nitrogen; (**b**) Frequency shift as a function of the hydrostatic pressure obtained from the FEM simulations for rigid clamp (black dotted line) and for soft clamp (red solid line). Lower inset: Image of the geometry employed in the simulation. Upper inset: Schematic of the radial expansion of the tube as a function of pressure (not to scale); (**c**) Schematic of the number of molecules inside the tube as a function of pressure (not to scale).

When the TMR device is full of nitrogen at atmospheric pressure and we apply a hydrostatic pressure (Δp) of 3 bar, the resonance frequency changes (Figure 2a). The resonance frequency of the TMR immediately responds to the pressure change, reaching a stationary value as soon as the pressure is stabilized (500 ms). Therefore, analogously to the density responsivity, we can define a pressure responsivity as $R_{hp}=\frac{\partial \left(\Delta f/f_{0}\right)}{\partial p}$. Under the experimental conditions, pressure responsivity has a value of −2.7 GPa^−1^ for nitrogen.

This hydrostatic pressure frequency response has its origin in a balance between mass and stiffness effects in the resonator. Therefore, it is possible to write the normalized frequency shift as $\frac{\Delta f}{f_{0}}={\left(\frac{\Delta f}{f_{0}}\right)}_{stiffnesss}+{\left(\frac{\Delta f}{f_{0}}\right)}_{mass}$.

Regarding the stiffness, the hydrostatic pressure applies a load on the inner channel wall (load effect) increasing the microchannel radius (momentum effect) [33]. This variation in the inner microchannel radius produces the change of the area moment of inertia. The inner radius linearly varies with the applied pressure as $\Delta R_{in}=\frac{R_{in}}{K}\Delta p$, being K the compressibility modulus of the fused silica wall (~37 GPa). Compressibility modulus can be defined as a function of Young’s modulus and Poison ratio (υ) as $K=\frac{E}{3\left[1-2\upsilon \right]}\;$ [34]. therefore, for small radius variations, the inertia term contribution to the mechanical frequency shift can be written as shown in Equation (3).

(3) $${\left(\frac{\Delta f}{f_{0}}\right)}_{momentum}=\frac{\Delta p}{K}$$

Additionally, the load applied by the pressure shifts the resonance frequency linearly with opposite sign [33], Equation (4).

(4) $${\left(\frac{\Delta f}{f_{0}}\right)}_{load}=-\frac{A_{i}}{2P_{E}}\Delta p$$

with $P_{E}$ being Euler’s critical buckling load and $A_{i}$ the area of the inner cannel.

The pressure responsivity of both combined effects is expected to be −0.319 GPa^−1^; however, given the polymeric clamps, there is a softening effect in the device [29]. The non-ideality of these clamps affects to pressure responsivity. To understand the role of soft clamp in pressure responsivity and checking the validity of the analytical model we have performed finite element simulations (FEM, COMSOL Multiphysics, Stockholm, Sweden) of an empty fused silica tube surrounded by SU-8 pads mimicking the geometry of the device (Figure 2b inset). In these simulations we have applied a constant pressure in the inner walls of the tube (hydrostatic pressure) while calculating its resonance frequency. Firstly, we constraint the displacement in the SU-8 pads (rigid clamp, black dotted line in Figure 2b), obtaining a linear response of −0.317 GPa^−1^, which perfectly agrees with the analytical model. Nevertheless, when we leave the structure to be free for deformation in the plane perpendicular to the TMR longitudinal axis (soft clamp, red solid line in Figure 2b), the pressure responsivity has a larger slope (−0.396 GPa^−1^). The difference between these slopes ($R_{clamp}$ = −0.079 GPa^−1^) can be attributed to strain effects in the soft clamp.

Despite the good agreement between analytical model and FEM simulations, stiffness effects cannot explain by themselves the pressure responsivity experimentally measured; therefore, we have to consider pressure-induced mass effects: fluid compressibility and tube expansion. As we have previously pointed out, the hydrostatic pressure produces the expansion of the inner radius (Figure 2b, inset). This radial expansion induces an added mass as the additional volume will be filled with more fluid. This added mass of the resonator shifts its resonance frequency as:

(5) $${\left(\frac{\Delta f}{f_{0}}\right)}_{volume}=-\frac{R_{\rho }\rho_{0}}{K}\Delta p$$

with $\rho_{0}$ being the fluid density at atmospheric pressure.

On the other hand, when a fluid is hydrostatically compressed, the number of molecules inside a certain volume increases (Figure 2c), which is translated into a density variation (Δρ) through the compressibility factor, β, as, $\Delta \rho =\rho_{0}\beta \Delta p$ [12]. This density variation is translated into an additional mass, consequently shifting the resonance to lower frequencies:

(6) $${\left(\frac{\Delta f}{f_{0}}\right)}_{compression}=-R_{\rho }\rho_{0}\beta \Delta p$$

In the experiments, we have the combination of the five aforementioned phenomena given a hydrostatic pressure responsivity ($R_{hp}$) resulting of the sum of the different slopes:

(7) $$R_{hp}=\frac{1}{K}-\frac{A_{i}}{2P_{E}}+R_{clamp}-\frac{R_{\rho }\rho_{0}}{K}-R_{\rho }\rho_{0}\beta$$

Given this analytical expression, the expected pressure responsivity for nitrogen is −2.6 GPa^−1^, which agrees with the experimental measurement, −2.7 GPa^−1^. Note that while stiffness terms (momentum, load and clamp) only depend on the device geometry, mass terms (volume and compression) depend on fluid properties, opening the door for a new source of information in suspended microchannel resonators.

## 4. Experimental Measurements

**Figure 3:**
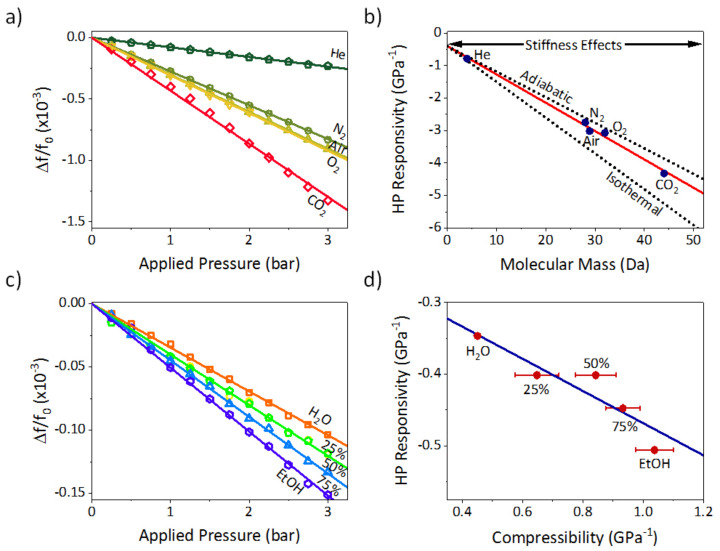
Fluid compressibility measurements. (**a**) Frequency shift measured as a function of the applied pressure for different gases: helium (pentagons), nitrogen (circles), oxygen (triangles), air (inverted triangles) and carbon dioxide (diamonds) and their linear fittings; (**b**) Hydrostatic pressure responsivity measured as a function of the molecular mass (solid circles) and its linear fit (solid line) for different gases. These data are compared with the expected trends for the analytical model (dotted lines); (**c**) Frequency shift measured as a function of the applied pressure for different concentration aqueous ethanol solutions: 0% (H_2_O, red squares), 25% (yellow circles), 50% (green pentagons), 75% (blue triangles) and 100% (EtOH, purple hexagons); (**d**) Hydrostatic pressure responsivity measured as a function of fluid compressibility (circles) and its linear fit (solid line).

To check the validity of the proposed model, we have measured the frequency shift induced by the hydrostatic compression of the inner fluid for different pressures and fluids at a constant laboratory temperature of 295 ± 1 K. For gases, the frequency shift as a function of applied pressure shows an inversely proportional dependency (Figure 3a), as expected from the analytical model. We have also measured the pressure responsivity for each gas by fitting the frequency shift data. When represented as a function of molecular mass, pressure responsivity also shows a linear dependency (Figure 3b), being in good agreement with an ideal gas model, in which, the parameter $\rho_{0}\beta_{T}$ can be written as follows:

(8) $$\rho_{0}\beta_{T}=\frac{m_{m}}{k_{B}T}$$

with $\beta_{T}$ being the isothermal compressibility factor, $m_{m}$ the molecular mass, $k_{B}$ being Boltzmann’s constant and T the temperature. Please note that for air, molecular mass has been calculated as an average value of the molecular masses of oxygen and nitrogen weighed to their concentrations (28.9 u). However, if the compression is adiabatic this compressibility factor has a different value, $\beta_{S}$, which can be obtained as $\beta_{S}=\beta_{T}/\gamma$. Being γ the heat capacity ratio (~1.4 for the gases used in this paper).

By fitting the pressure responsivity as a function of the molecular mass we obtain a slope of −0.087 ± 0.004 GPa^−1^·Da^−1^. This value is amid the value expected for an ideal gas isothermal compression (−0.110 GPa^−1^·Da^−1^) and an ideal gas adiabatic compression (−0.079 GPa^−1^·Da^−1^). Ideally, the gas compression should be adiabatic; nevertheless, the inner microchannel is not perfectly isolated, producing this intermediate case. Moreover, this linear fitting reveals an offset of 0.4 ± 0.1 GPa^−1^, which perfectly agrees with the stiffness effects expected from the analytical model. Considering a limit of detection of 1.3 × 10^−3^ GPa^−1^ in pressure responsivity, this device can detect changes in molecular mass with a resolution of 0.01 Da.

We repeat the same experiment but filling the TMR device with aqueous ethanol solutions of different volumetric concentrations from 0% to 100%. Frequency shift as a function of hydrostatic pressure also presents a linear dependency, being the more compressible liquids those in which the frequency shift is larger (Figure 3c). This behavior is again in very good agreement with the analytical model. We plot hydrostatic pressure responsivity versus compressibility (Figure 3d). As we know from the gas experiments, hydrostatic compressions in this setup are an intermediate case between isothermal and adiabatic compression; therefore, we choose as compressibility value the arithmetic average of the isothermal and adiabatic compressibility factor for each solution from its nominal values [12,35]. By linear fitting of this data, we obtain an offset of and slope of −0.22 ± 0.04, resulting in good agreement with the values predicted in the analytical model (−0.17). The experimental data dispersion may have a thermodynamic origin as thermal conductivity is different for each solution. This dispersion in thermal conductivity may result in the compression process being closer to adiabatic or isothermal, depending on its concentration.

## 5. Conclusions

In this work we have both theoretically predicted and experimentally demonstrated the effects of the hydrostatic pressure in the resonant behavior of a suspended microchannel resonator. We have demonstrated that, additionally to the stiffness terms, it is necessary considering mass effects (compressibility) to explain the measured values of resonance frequency shift in suspended microchannel resonators. Stiffness terms are only determined by the geometry of the device, making them suitable for pressure sensing; nonetheless, when pressure-induced mass effects become comparable to stiffness effects, pressure response also depends on fluid properties. Consequently, we have demonstrated that suspended microchannel resonators can be used to measure fluid density with a resolution of 0.7 µg/mL when working with aqueous solutions, one order of magnitude better than the current state-of-the-art, and compressibility which, for gases, can be related to molecular mass obtaining a resolution of 0.01 Da. These results open the door to use suspended microchannel resonators as non-specific gas sensors. The obtained mass resolution corresponds to the difference in compressibility of pure air to that of 50 ppm of radon in air. Compressibility measurements shown in this work can be combined with mass density and refractive index measurements given by transparent microcapillary resonators for fluid characterization.
